# Intranodal palisaded myofibroblastoma: a case report from an unusual site

**DOI:** 10.11604/pamj.2015.22.78.7884

**Published:** 2015-10-01

**Authors:** Laraqui Hicham, Mouna Khmou, Mohammed Najih, Fouad Zouaidia, Khalid Sair, Basma El Khannoussi

**Affiliations:** 1 Department of Surgery 2, Mohammed V Military Hospital, Rabat, Morocco; 2 Department of Pathology, National Institute of Oncology, Rabat, Morocco; 3 Department of Pathology, Ibn Sina University Hospital, Rabat, Morocco; 4 Department of Surgery 1, Mohammed V Military Hospital, Rabat, Morocco

**Keywords:** Intranodal palisaded myofibroblastoma, retroperitoneum, lymph node

## Abstract

Intranodal palisaded myofibroblastoma is a rare lymph node benign tumor, of unknown pathogenesis. Although benign, this lesion is frequently confused with metastatic lesions, especially in atypical sites. We report a 39-year-old man with a history of testicular malignant mixed germ cell tumor, presented with abdominal painless mass. The computed tomography of the abdomen confirmed the presence of 180 × 140 mm2 mass in the retroperitoneum with lympadenopathy on the right measuring 20 x 15 mm. The patient underwent exploratory laparotomy, and a surgical exerese of the retroperitoneeum lymph node was made. Histological and immunohistochemical examination confirmed the diagnosis of intranodal palissaded myofibroblastoma. This entity has been previously described, only once, in retroperitoneal region. Despite to the rarity of this neoplasm, we discuss clinicopathologic features and differential diagnosis.

## Introduction

Intranodal palisaded myofibroblastoma is a benign intanodal mesenchymal proliferation composed of myofibroblastic cells often with focal nuclear palisading, intralesional hemorrhage, and collagen with stellate extensions. Intranodal hemorragic spindle cell tumor with amiantoid fibers is synonymous. The diagnosis is only based on microscopic and immunohistochemical features wich can differentiated it from other mesenchymal tumors. In this paper we report another case of IPM, but originating from retroperitoneum. Apart from the rarity of this tumour, we also discussed its characteristic features, pathogenesis and differential diagnosis.

## Patient and observation

A 39-year-old man with a history of testicular malignant mixed germ cell tumor, presented with 6 months history of abdominal mass, which was rapidly growing. Physical examination revealed a firm nontender mass in the left hypochondriac region. The computed tomography confirmed the presence of 180 × 140 mm^2^ mass in the retroperitoneum, separate from the small intestinal and the psoas muscle without any evidence of local infiltration with lympadenopathy on the right location measuring 20 x 15 mm. The patient underwent exploratory laparotomy, and a surgical excision of the lymph node was made. The patient was discharged without complications.

**Figure 1:**
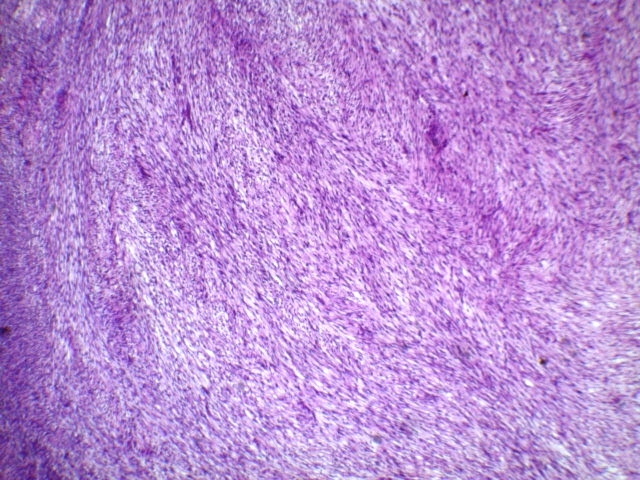
Low power view of the tumour with interlacing fascicles of spindle cells

**Figure 2:**
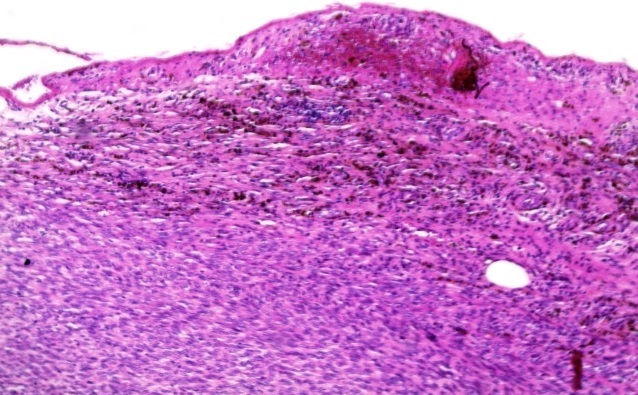
High power view of the tumour with hemosiderin pigment and lymphoid cell with irregular distribution observed among the spindle cells

**Figure 3:**
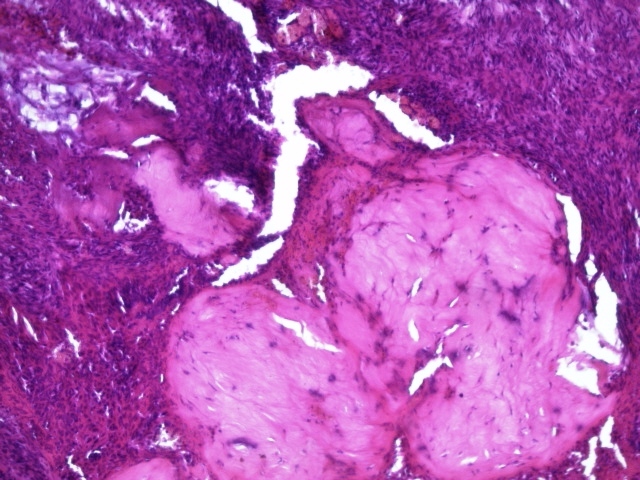
High power view of the tumour showing short fascicles of spindle cells and amianthoid fibres

**Figure 4:**
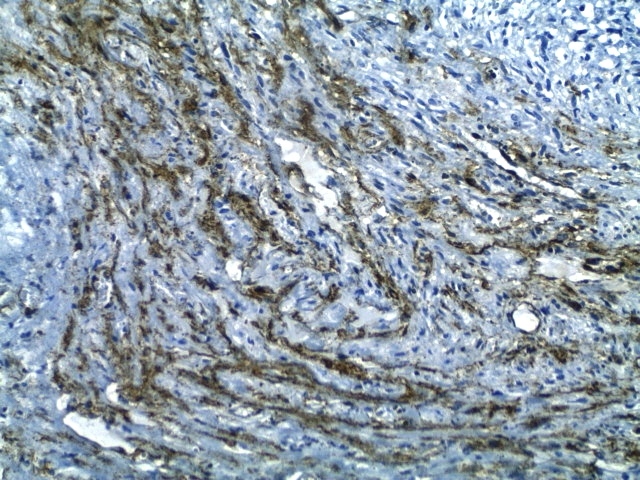
High power view of the tumour showing strong expression of Smooth muscle actin (SMA)

Grossly, two firm fragments were received. On slicing, white and solid areas, alternating with haemorrhagic areas, were noted. Microscopic examination showed a spindle-cell proliferation with variable cellularity. The cells formed short interlacing fascicles, and had slightly wavy nuclei displayed a patchy pattern of palisading (Figure 1). Those cells were observed to have eosinophilic cytoplasm, and indistinct borders. Collagen accumulations were easily recognized as “amianthoid fibers” in some areas (Figure 2). In the peripheral portion of the tumor, reactive lymphoid, hemosiderin-laden macrophages, and extravasated erythrocytes were also noted (Figure 3). The mitotic rate was estimated at three per 10 hpf. No atypia, or necrosis was identified. Immunohistochemical analysis revealed positivity for Smooth muscle actin (SMA) (Figure 4) and negativity for Desmin, S-100 protein, CD117, CD34, cytokeratin, and EMA. Based on these results, the diagnosed of “intranodal palisaded myofibroblastoma” was made.

## Discussion

Intranodal palisaded myofibroblastoma (IPM) is a rare benign mesenchymal intranodal proliferation composed of myofibroblastic cells [1]. The process usually involves a solitary groin lymph node, involvement of submandibular/neck, mediastinum has been described. The peritoneum location is extremly rare with only one case reported in the litterature [2]. IPM was initially describded by Deligdish and Katz as neurilemmoma or schwannoma and later classified as palisaded myofibroblastoma by Weiss [3, 4].

IPM affects patients range from 19-78 years of age, with a peak incidence in the sixth decade. The male to female ration is approximately 3 to 2 without specific race [5]. The clinical presentation is generally a slow growing mass with paineless [2]. In the retroperitoneum case reported, pain with right flank mass was the principal symptoms. In our case, this tumor was discovered fortunatly as lymphadenopathy.

Gross examination reveals a 0,6 to 6,0 cm, well circumscribed, rubbery to firm mass that on cut section is gray-white with brown hemorragic foci. In some cases, a residual white lympoid tissu may be present at the periphery. Histologic examination demonstrates an intranodal, spindle cell proliferation arranged in sheets and short intersecting fasciles with focal palisading. Areas of hemorrhage and hemosiderin deposition are usually present. There are usually scattered islands of collagen with peripheral stellate or starbust-like extensions resembling to amiantoid fibers. Mitotic activity is typically low [6, 7]. The spindle cells of IPM are immunoreactive for actin and nonreactive for desmin, S-100 protein, glial fibrillary acidic protein (GFAP), synaptophysin, EMA and keratin cocktail. Examined cases have been negative for human herpesvirus (HHV)-8 or Epstein Barr Virus (EBV) polymerase chain reaction products [5]. This immunoprofile supports the notion that IPM is probably arising from myofibroblast or smooth muscle cell of the lymph node blood vessels [3].

The most differential diagnosis for IPM includes schwannoma, Kaposi sarcoma, gastrointestinal stromal tumor, spindle cell melanoma, and metastatic spindle cell sarcoma.

The clinical history, examination and typical histological characteristics help in the correct diagnosis of the IPM [8]. Intranodal Schwannoma has a predilection for sinusoidal regions of the lymph node and generally exhibits more nuclear atypia with a higher mitotic rate than IPM, the tumor cells are positive for S100 protein. Kaposi Sarcoma shows numerous slit-like vascular spaces, extravasation of red blood cells, grape-like clusters of eosinophilic hyaline bodies and nuclear immunoreactivity for HHV8 [8]. Nodal sarcoma metastases are uncommon and typically feature much more atypia and mitotic activity than are seen in IPM. Furthermore, spindle cell melanoma can be differentiated from IPM by high proliferative activity and marked cellular atypia. Spindle cell melanoma is also positive for S100 and HMB-45 [9]. The gastrointestinal stromal tumor is positive for CD117/C- kit while IPM is negative [2].

For treatment, simple local excision is the treatment of choice, with benign clinical course and very low or negligible rate of recurrence [10].

## Conclusion

IPM in the retroperitoneum region is an original case and will be the second case in the literature. This report raises questions about etiology and pathogenesis of this tumor. IPM should be kept in mind for differential diagnosis in patients presenting an abdominal mass in the retropreitoneal region.
